# Supplementary material for: Tofacitinib May Inhibit Myofibroblast Differentiation from Rheumatoid-Fibroblast-like Synoviocytes Induced by TGF-β and IL-6
Source: Pharmaceuticals (Basel). 2022 May 18;15(5):622. doi: 10.3390/ph15050622 (PMC9147406; doi:10.3390/ph15050622)
Supplement: Supplementary file 1 [file pharmaceuticals-15-00622-s001.zip › pharmaceuticals-1645378-supplementary.pdf]

Supplementary Materials

Supplementary Table S1. Demographic and clinical characteristics of patients with RA and HCs. RA, Rheumatoid Arthritis; HC–healthy controls; DAS28–Disease Activity Score on 28 joints; ACPA–anti citrullinated peptide antibody; RF–rheumatoid factor. NA–not applicable.

	**RA (7)**	**HC (7)**
**Age at the time of biopsy (median years, range)**	56 (45-61)	58 (45-63)
**Gender (n, % of female)**	6 (86%)	6 (85%)
**Disease duration (median years, range)**	3 (1-5)	NA
**ACPA positivity (n, %)**	10 (100%)	NA
**RF status (n, %)**	10 (100%)	NA
**DAS28 (median, range)**	5.1 (4.8 – 5.9)	NA
**Low dosage of steroids (n, %)**	8 (80%)	NA
**Methotrexate (n, %)**	10 (100%)	NA

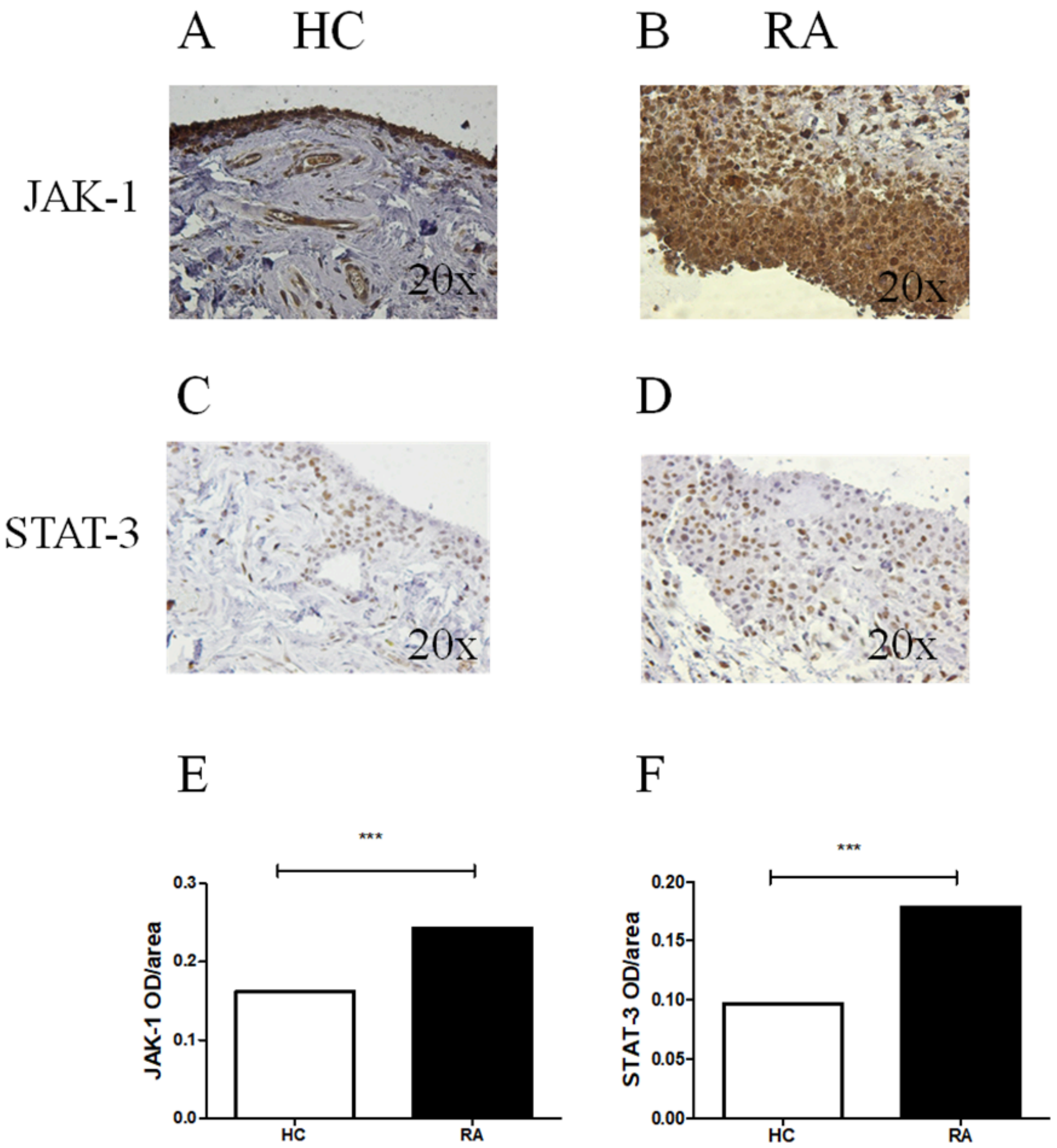

Supplementary Figure S1. JAK-1 and STAT-3 expression in synovial tissue of patients with RA and HCs. (A-B) JAK-1 IHC of HC (A) and RA (B) synovial tissues; (C-D) STAT-3 IHC of HC (C) and RA (D) synovial tissue; (E-F) In RA synovial tissue, the optical density (OD) of JAK-1 (E) and STAT-3 (F) was significantly increased when compared to HC. The histogram showed median for each synovial tissue (***= p≤0.0003). Original magnification X20.

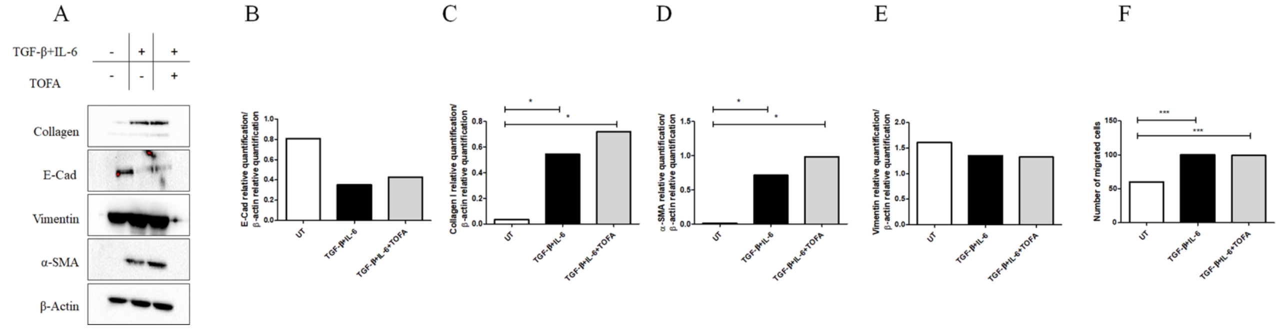

Supplementary Figure S2. Western blot analyses of Collagen I, E-Cad, Vimentin and α-SMA.

A) Western blot analyses of Collagen I, E-Cad, Vimentin and α-SMA, pictures are representative of all the experiments. The grouping of gels/blots cropped from different parts of the same gel. No high-contrast (overexposure) of blots was performed.

(B-E) The densitometry analysis of western blot shows that TGF-β+IL-6 stimulation of RAFLSs induced a significant increase of Collagen I and α-SMA. However, the tofacitinib (TOFA) administration was unable to prevent TGF-β+IL-6 effects. The histogram showed median of triplicate experiments (*= $p=0.049$).

F) Migrated cells [number of migrated cells after stimulation with TGF-β+IL-6 100 (89-145) vs number of migrated UT-cells 60 (45-87), $p=0.0004$]. The histogram showed the median of migrated RA-FLSs. TGF-β+IL-6 significantly induced an increase of number of migrated FLSs, when compared untreated (UT) cells and the treatment with tofacitinib was unable to prevent the effects of TGF-β+IL-6 (***= $p\leq0.006$).
